# Placental Pathology and Maternal Risk Factors for Stillbirth: A Case-Control Study

**DOI:** 10.7759/cureus.39339

**Published:** 2023-05-22

**Authors:** Ojaswini Patel, Pranati Pradhan, Prerana Das, Sanjeeb K Mishra

**Affiliations:** 1 Obstetrics and Gynaecology, Veer Surendra Sai Institute of Medical Sciences and Research, Sambalpur, IND; 2 Field Epidemiology Training Program, Indian Council of Medical Research, Chennai, IND; 3 Community Medicine, Veer Surendra Sai Institute of Medical Sciences and Research, Sambalpur, IND

**Keywords:** stillbirth, case-control study, placental pathology, risk factor, high-risk pregnancy

## Abstract

Background

Fetal death is the delivery of a fetus with no sign of life, as indicated by the absence of breathing, heartbeat, pulsation of the umbilical cord, or definite movement of voluntary muscles. Nearly 2.6 million stillbirths are estimated to occur worldwide every year. Almost all of these (98%) stillbirths occur in low- and middle-income countries. About one-sixth of the stillbirths globally were recorded in India in 2019, making it the most burdened country in the world. In light of this, we conducted a study to identify the placental pathologies and maternal factors associated with stillbirth.

Methodology

A case-control study was conducted at the Department of Obstetrics & Gynecology, Veer Surendra Sai Institute of Medical Sciences and Research (VIMSAR), from June 2022 to May 2023. Cases included pregnant women with a gestational age of 28 weeks or more who delivered a stillbirth infant at VIMSAR, and controls included gestational age-matched deliveries with live birth. Consent to participate in the study was obtained before enrolment. The final sample size was 79 cases and controls. The chi-square test was performed for bivariate analysis, and logistic regression was used for multivariate analysis.

Results

In our study, we found a significant association between maternal age of more than 30 years (odds ratio (OR) = 3.01, 95% confidence interval (CI) = 1.91-4.22, p = 0.012), maternal education (with up to the primary level or less: OR = 6.19, 95% CI = 2.92-7.87, p = 0.012), history of addiction (tobacco chewing: OR = 5.58, 95% CI = 3.71-7.11, p = 0.03), and the number of antenatal visits (no visit: OR = 6.87, 95% CI = 2.91-7.79, p = 0.019) with an increased risk of stillbirth. Among the obstetrical complications, pre-eclampsia/eclampsia (OR = 3.87, 95% CI = 1.98-5.11, p = 0.001), premature rupture of membranes (PROM)/preterm premature rupture of the membranes (PPROM) (OR = 2.49, 95% CI = 1.31-3.91, p = 0.03) and antepartum hemorrhage (APH) (OR = 2.66, 95% CI = 1.65-3.58, p = 0.02) were found to be significantly related with stillbirth. Among placental pathologies, uteroplacental vascular pathology (OR = 7.39, 95% CI = 3.01-8.97), acute chorioamnionitis (OR = 3.35, 95% CI = 2.11-5.21), chronic inflammation (OR = 2.33, 95% CI = 1.91 4.17), calcific changes (OR = 4.46, 95% CI = 2.56-6.01), and retroplacental clots (OR = 9.95, 95% CI = 4.39-11.71) were associated with stillbirth.

Conclusions

In our study, advanced maternal age, absence of antenatal visits, low level of education, tobacco addiction, pre-eclampsia/eclampsia, APH, and PROM in pregnancy were the major risk factors associated with stillbirth. Uteroplacental vascular pathology, chorioamnionitis, chronic inflammation, retroplacental hematoma, and calcific changes were the most significant placental lesions associated with stillbirth.

## Introduction

Stillbirth is defined as “Fetal death prior to complete expulsion or extraction from the mother of a product of human conception irrespective of the duration of pregnancy and which is not an induced termination of pregnancy. The death is indicated by the act that after such expulsion or extraction, the fetus does not breathe or show any other evidence of life such as the beating of the heart, pulsation of the umbilical cord, or definite movement of voluntary muscles” [1,2]. Nearly 2.6 million stillbirths are estimated to occur worldwide every year. Almost all of these (98%) stillbirths occur in low- and middle-income countries (LMICs) [3]. About one-sixth of global stillbirths were recorded in India in 2019, making it the most burdened country in the world [3]. India has intervened effectively to substantially reduce the stillbirth rate over the past two decades. The rate had declined from 29.6 in 2000 to 13.9 stillbirths per 1,000 births in 2019, a reduction of more than 50%. Globally, a reduction in the stillbirth rate was recorded at about 35% during this period [4].

As of 2019, more than one-third of the global burden of stillbirths was concentrated in India (17.3%), Pakistan (9.7%), and Nigeria (8.7%) [5,6]. After the introduction of the Newborn Action Plan (INAP), there occurred a steady decline in stillbirths with a stillbirth rate of 13.4 (4.2-24.2), 13.1 (4.2-22.2), and 12.4 (3.7-22.5) in 2017-2018, 2018-2019, and 2019-2020, respectively [7,8]. In India, Odisha, Madhya Pradesh, Rajasthan, and Chhattisgarh form a contiguous east-west belt of high stillbirth rate [9].

According to the Stillbirth Collaborative Research Network of the National Institute of Child Health and Human Development, the common causes of stillbirth are divided into eight categories as obstetrical complications (29%), placental abnormalities (24%), fetal malformations (14%), infections (13%), umbilical cord abnormalities (10%), hypertensive disorders (9%), medical complications (8%), and undetermined (24%). In the report, antepartum hemorrhage (APH) in the present pregnancy was significantly associated with stillbirth. It had 2.66 times higher odds of developing stillbirth than those without APH. Hypertensive disorders in pregnancy (pre-eclampsia/eclampsia) were associated with higher odds of developing stillbirth in pregnancy than those without pre-eclampsia/eclampsia [3].

Although India has made substantial progress in reducing maternal and child mortality rates over the past two decades, the high rate of stillbirths continues to be a public health problem. It has not received the needed attention as evidenced by the non-inclusion as a specific target in the Sustainable Development Goals (SDGs) [6]. In light of this, this study aimed to determine the placental pathologies and maternal factors associated with stillbirths at Veer Surendra Sai Institute of Medical Sciences and Research (VIMSAR). The knowledge gained may help identify preventive measures that would lower stillbirth rates.

## Materials and methods

A case-control study was conducted in the Department of Obstetrics & Gynecology, VIMSAR, from June 2022 to May 2023. Ethical approval for this study was obtained from the VSS Institutional Ethics Committee (approval number: 201-2022).

Inclusion and exclusion criteria

Pregnant women with a gestational age of 28 weeks or more who delivered a stillbirth infant were recruited as cases in this study. Pregnant women with a gestation age of 28 weeks or more who delivered a live infant during the study period were recruited as controls. The gestational age was the only matching criterion used in the study. For each recruited case, a control was selected within 24 hours. Infants with apparent congenital anomalies were excluded from the study.

Study variables

The variables included in the study were age, maternal education, marital status, employment status, number of antenatal care (ANC) visits, maternal addiction (daily consumption of tobacco products or alcohol), parity, previous history of stillbirth, APH, premature rupture of membranes (PROM), pre-eclampsia, medical history, and placental pathologies.

Statistical analysis

Data were entered and cleaned in Microsoft Excel. Continuous variables are presented as mean and standard deviation (SD). Categorical variables are presented as percentages. The chi-square test was performed to test association, and a p-value of less than 0.05 was considered significant. Multivariate logistic regression was performed to determine the association of maternal factors with stillbirths after checking for multicollinearity and interaction in R package version 4.2.2.

## Results

Our study included 79 cases and an equal number of controls. Most women in both cases (51) and controls (66) belonged to the 21-29-year age group. The mean age was 25.4 ± 4.2 years in the control group compared to 28.5 ± 6.7 years in the cases. The mean body mass index (BMI) was 23.1 ± 4.1 kg/m^2^ in the control group compared to 23.6 ± 5.3 kg/m^2^ in cases. Concerning educational status, most women were educated up to high school (cases = 57, controls = 60). The case group had seven unmarried pregnancies compared to five in the control group. Addiction to tobacco or alcohol was seen in 10 cases and two controls (Table 1). On logistic regression analysis, advanced maternal age, poor socioeconomic status, and positive history of addiction were significantly associated with increased stillbirths.

**Table 1:** Sociodemographic characteristics of pregnant women seeking obstetrics care at Veer Surendra Sai Institute of Medical Sciences and Research (VIMSAR), Burla (n = 158).

Characteristics		Controls (n = 79), n (%)	Cases (n = 79), n (%)
Age (years)	20 or less	4 (5)	7 (9)
	21–29	66 (83.5)	51 (64.5)
	30 or more	9 (11.5)	21 (26.5)
Education	Primary or below (up to 7th class)	8 (10)	18 (23)
	High school (8th to 10th class)	60 (76)	57 (72)
	College or above (11th class and above)	11 (14)	4 (5)
Marital status	Married	74 (93.7)	72 (91)
	Unmarried	5 (6.3)	7 (9)
Employment	Employed	68 (86)	64 (81)
	Unemployment	11 (14)	15 (19)
History of addiction	No	77 (97.5)	69 (87.3)
	Yes	2 (2.5)	10 (12.7)

Mothers with APH had 2.66 times higher odds of developing stillbirth than those without APH. Pre-eclampsia/eclampsia had 3.87 times higher odds of developing stillbirth in pregnancy than without pre-eclampsia/eclampsia. PROM/preterm premature rupture of membranes (PPROM) had 2.49 times higher odds of developing stillbirth in the present pregnancy than those without membrane rupture (Table 2).

**Table 2:** Obstetric characteristics of pregnancies resulting in stillbirths (n = 79) compared with live births (n = 79) among women seeking care at Veer Surendra Sai Institute of Medical Sciences and Research (VIMSAR), Burla. ANC: antenatal care; APH: antepartum hemorrhage; PROM: premature rupture of membranes; PPROM: preterm premature rupture of membranes

Characteristics		Controls (n = 79), n (%)	Cases (n = 79), n (%)	Odds ratio	P-value
History of stillbirth	No	68 (86)	56 (71)	1.00	0.023
	Yes	11 (14)	23 (29)	2.54	
Parity	Primi	54 (68.3)	47 (59.5)	1.00	-
	Multi	24 (30.4)	27 (34.2)	1.29	0.456
	Grand Multi	1 (1.3)	5 (6.3)	5.70	0.116
ANC visits	0	2 (2.5)	8 (10)	6.87	0.019
	1-3	24 (30.4)	40 (51)	2.85	0.002
	4 or more	53 (67.1)	31 (39)	1.00	-
APH	No	69 (87.3)	57 (72.2)	1.00	0.0201
	Yes	10 (12.7)	22 (27.8)	2.66	
Pre-eclampsia	No	69 (87.3)	47 (40.5)	1.00	0.001
	Yes	10 (12.7)	32 (59.5)	3.87	
PROM/PPROM	No	69 (87.3)	58 (73.4)	1.00	0.0306
	Yes	10 (12.7)	21 (26.6)	2.49	

Anemia in pregnancy had 2.19 times higher odds of developing stillbirth than those without anemia. Diabetes, HIV, and malaria were not found to have statistically significant correlations with stillbirth (Table 3).

**Table 3:** Medical conditions associated with stillbirths among women seeking care at Veer Surendra Sai Institute of Medical Sciences and Research (VIMSAR), Burla (n = 158). BMI- Body mass index, HIV - Human immunodeficiency virus

Characteristics		Controls (n = 79), n (%)	Cases (n = 79), n (%)	Odds ratio	P-value
Body mass index (kg/m^2^)	18–24	67 (84.8)	63 (79.8)	1.00	-
	25–29	7 (8.9)	10 (12.6)	1.52	0.42
	30 or more	5 (6.3)	6 (7.6)	1.27	0.69
Diabetes mellitus	No	77 (97.5)	70 (88.6)	1.00	0.085
	Yes	2 (2.5)	9 (11.4)	3.26	
Anemia	No	58 (73.4)	44 (55.7)	1.00	0.021
	Yes	21 (26.6)	35 (44.3)	2.19	
HIV status	No	74 (94)	77 (97.5)	1.00	0.26
	Yes	5 (6)	2 (2.5)	0.38	
Malaria	No	76 (96)	75 (95)	1.00	0.70
	Yes	3 (4)	4 (5)	1.35	

Placental pathologies included chronic inflammations, uteroplacental vascular pathology, acute chorioamnionitis, cord edema, coagulation-related lesion, calcific changes, and retroplacental clots. Although all placental pathologies indicated a positive association with stillbirth, but only five were statistically significant. These included uteroplacental vascular pathology (OR (95% CI) = 7.39), acute chorioamnionitis (OR (95% CI) = 3.35), chronic inflammation (OR = (95% CI) = 2.33), calcific changes (OR = (95% CI) = 4.46), and retroplacental clots (OR = (95% CI) = 9.95) (Table 4).

**Table 4:** Association of different placental pathologies with stillbirth.

Placental pathology	Controls (n = 79)	Cases (n = 79)	Odds ratio	P-value
Uteroplacental vascular pathology	12	45	7.39	<0.0001
Acute chorioamnionitis	4	12	3.35	0.044
Chronic inflammation	10	18	2.33	0.0489
Coagulation-related lesions	4	8	2.11	0.238
Cord edema	1	2	2.02	0.567
Calcific changes	10	31	4.46	0.0003
Retroplacental clots	4	30	9.95	<0.0001

## Discussion

Our study found that advanced maternal age significantly contributes to stillbirths. Maternal age of more than 30 years had an OR of 3.01 with a p-value of 0.012. Women of advanced maternal age are at a higher risk of maternal hypertensive disorders, gestational diabetes, and other complications, which, in turn, are associated with the risk of stillbirth. Waldenström et al. found that advanced maternal age is an independent risk factor for stillbirth in nulliparous women. They reported that stillbirth rates increase synchronously with maternal age [10]. In first births, the chance of stillbirth increases with the age of the mother. This increase was almost 25% at 30-34 years of age compared to age 25-29 and doubled at age 35 [11]. Huang et al. found that women with advanced maternal age have an increased risk of stillbirth [11]. Additionally, Reddy et al. showed that women over the age of 35 had a greater chance of stillbirth during the entire gestation, with the highest risk occurring between weeks 37 and 41. The relative risk of stillbirth was 1.32 (95% CI = 1.22-1.43) for women aged 35 to 39 years and 1.88 (95% CI = 1.64-2.16) for women aged 40 years or older at 37 to 41 weeks compared to women under the age of 35 years [12].

This study suggested a significant association between maternal education, history of addiction, and the number of prenatal visits, with fewer visits having a higher risk of stillbirth. Similar findings were reported by Auger et al. who concluded that low education is associated with stillbirth throughout gestation. In their study, low education was most strongly associated with a stillbirth at ≥28 weeks relative to higher education [13]. Altijani et al. found that indicators of socioeconomic deprivation (female illiteracy), history of addiction (tobacco chewing), and adverse pregnancy and birth characteristics (fewer ANC visits) were associated with stillbirth [14]. In this study, a previous history of stillbirth increased the risk of stillbirth. Similar findings were reported by another study from Africa [15]. A systematic review study conducted by the Lamont et al. found that a history of stillbirth increased the risk of recurrence [16]. A history of stillbirth was reported as a major risk factor for subsequent loss, particularly in the early stages of pregnancy (22-28 weeks), according to different studies. Compared to women who had a live delivery in their first pregnancy, women with past stillbirth have a two-fold increased risk of recurrence [17]. To reduce the risk of perinatal mortality, women who have previously experienced a stillbirth should be advised against elective induction in their following pregnancies at 37-38 weeks of gestation. The necessity for vigilant monitoring of these susceptible women in subsequent pregnancies is highlighted by the significant link between the positive history of stillbirths and high recurrences.

Among the obstetrical complications, pre-eclampsia/eclampsia, PROM/PPROM, and APH were found to have statistical evidence of stillbirth. Ngwenya et al. reported that women with severe pre-eclampsia are at significantly increased risk of stillbirth with a prevalence of 9.8% [18]. Our study is also similar to the study done to classify causes of stillbirth for six LMICs using a prospectively defined algorithm. McClure et al. suggested that the primary cause of stillbirth was fetal asphyxia (46.6%) associated with prolonged or obstructed labor (38%), pre-eclampsia/eclampsia (18%)c, and APH (19%) [19]. O'Leary et al. concluded that fetomaternal hemorrhage is a major contributor to antepartum stillbirth [20].

Our research showed that placenta abnormalities were substantially more prevalent in stillbirths than in live births. Uteroplacental vascular pathology, acute chorioamnionitis, chronic inflammation, calcific alterations, and retroplacental clots were the placental abnormalities that were substantially linked. The findings of this study are consistent with those of a comprehensive analysis performed in the United Kingdom by Ptacek et al. who noted many placental diseases linked to stillbirths, including endovasculitis, fetal thrombotic vasculopathy, cord anomalies, and delayed villous maturation [21]. They also reported inflammatory abnormalities such as villitis, chorioamnionitis, and endovasculitis associated with stillbirth. The classification system used affects the utility of histopathological examination of the placenta. International consensus is required for diagnostic criteria and terminology to describe placental abnormalities and the classification of stillbirths [21]. According to our research, early preterm birth and stillbirth are both frequently accompanied by acute chorioamnionitis and other inflammatory diseases. A rise in placenta infections connected to the causative disease that triggers labor and results in stillbirths and premature births may be the cause of this. A similar study from the United States found that infection is an important cause of stillbirths worldwide. In LMICs, 50% of stillbirths or more are probably caused by an infection [22].

Study limitations

This study had some limitations. Exclusion criteria were based only on a gross assessment of congenital malformations. Because no genetic or molecular testing was performed for diseases such as Down’s syndrome or other aneuploidies, some of the stillbirths included in this study may have had genetic disorders that may have affected the investigation of the placental pathology. Second, there was a lack of information on microbiologic testing to identify illnesses that are frequently identified during pregnancy, such as the TORCH infection, which may have caused the placenta to undergo major alterations.

## Conclusions

We observed that uteroplacental vascular pathology, chronic inflammation, chorioamnionitis, calcific changes, and retroplacental hematoma were the most significant placental lesions associated with stillbirth. Significant risk factors for stillbirth were maternal medical problems and obstetric features, advanced maternal age, fewer prenatal visits, history of stillbirths, pre-eclampsia/eclampsia, APH, PROM/PPROM, and anemia throughout pregnancy. Adequate prenatal counseling and regular ANC attendance are recommended as they allow screening for potential risk factors for stillbirth and educating women, hence ensuring successful pregnancy outcomes. A minority of cases are associated with specific placental pathologies, often with high recurrence rates, which can be diagnosed only on microscopic examination of the placenta. The results of this study may pique pathologists’ and obstetricians’ curiosity about the placenta in the stillbirth population, which would shed light on the etiology of stillbirth. To further identify the precise causes of stillbirth, more research is required on the factors that influence stillbirth, such as postmortem statistics, genetic and molecular tests, microbiology, and fetal blood/urine culture.
